# Dysregulation of Stemness Pathways in HPV Mediated Cervical Malignant Transformation Identifies Potential Oncotherapy Targets

**DOI:** 10.3389/fcimb.2020.00307

**Published:** 2020-06-25

**Authors:** Megha Budhwani, Samuel W. Lukowski, Sandro V. Porceddu, Ian H. Frazer, Janin Chandra

**Affiliations:** ^1^Diamantina Institute, Translational Research Institute, The University of Queensland, Woolloongabba, QLD, Australia; ^2^Institute for Molecular Bioscience, The University of Queensland, St Lucia, QLD, Australia; ^3^Cancer Services, Princess Alexandra Hospital, Woolloongabba, QLD, Australia; ^4^Faculty of Medicine, The University of Queensland, Brisbane, QLD, Australia

**Keywords:** HPV, cervical cancer, cancer stem cells, Notch, Wnt, Hedgehog, Hippo, Focal Adhesion

## Abstract

Human papillomavirus (HPV) infection is associated with a range of malignancies that affect anogenital and oropharyngeal sites. α-HPVs dominantly infect basal epithelial cells of mucosal tissues, where they dysregulate cell division and local immunity. The cervix is one of the mucosal sites most susceptible to HPV infections. It consists of anatomically diverse regions, and the majority of cervical intraepithelial neoplasia and cancers arise within the cervical squamo-columnar junction where undifferentiated basal progenitor cells with stem cell properties are found. The cancer stem cell theory particularly associates tumorigenesis, invasion, dissemination, and metastasis with cancer cells exhibiting stem cell properties. In this perspective, we discuss evidence of a cervical cancer stem cell niche and explore the association of stemness related genes with 5-year survival using a publicly available transcriptomic dataset of a cervical cancer cohort. We report that poor prognosis in this cohort correlates with overexpression of a subset of stemness pathway genes, a majority of which regulate the central Focal Adhesion pathway, and are also found to be enriched in the HPV infection pathway. These observations support therapeutic targeting of stemness genes overexpressed by mucosal cells infected with high-risk HPVs.

## Introduction to HPV Virology and Disease

Oncogenic human papillomavirus (HPV) infections are responsible for 5% of the global human cancer burden, resulting in over 634,000 new cancers annually, of which ~90% are in women (Schiffman et al., 2016). Intraepithelial neoplasia and cancers driven by oncogenic HPV infections include those of the cervix, vagina, vulva, penis, and anus, and of the oropharynx where 570,000 new cases of cervical cancer alone and associated 311,000 female deaths were reported in 2018 (Arbyn et al., 2020). HPV infection is also associated with a proportion of bladder cancer (Zapatka et al., 2020). Five genera of HPVs (alpha, beta, gamma, mu, nu) are classified, comprising more than 450 HPV genotypes, but only so called high-risk α-HPVs (HPV16, 18, 31, 33, 35, 39, 45, 51, 52, 56, 58, 59, and 66) are clearly associated with malignancy, and have been classified as group I carcinogens (Burley et al., 2020). α-HPV types 16 and 18 account for 70% of cervical cancers (Tommasino, 2014) while low-risk genotypes are identified in mucosal and cutaneous papillomas (warts) and in apparently normal skin (Devitt et al., 2019) and genital epithelium (Egawa et al., 2015).

HPV16 is an epitheliotropic 60 nm small, non-enveloped, 8-kb circular double-stranded DNA virus and infects basal keratinocytes primarily of mucosal epithelium, where viral DNA is maintained within the nucleus as episomal DNA (Zhou C. et al., 2019). HPV genes include early (E1–E7) non-structural and late (L1-2) structural genes. In proliferating basal cells, the viral DNA is amplified to 50–100 copies, while viral gene expression is limited to low levels of E1 and E2 (Moody and Laimins, 2010). Viral early and late gene expression, however, increases in differentiating keratinocytes. As HPV uses the host's DNA replication machinery to replicate its episome, it utilizes strategies to delay basal keratinocyte differentiation and exit from cell cycle. HPV proteins E6 and E7 prolong keratinocyte survival and proliferation by initiating p53 degradation and continued E2F release (Yim and Park, 2005; Yeo-Teh et al., 2018), thereby enabling HPV episomal replication. L1 and L2 capsid proteins are expressed in terminally differentiated cells, where they assemble into functional viral particles and are shed from the surface (Egawa et al., 2015). Malignant transformation of HPV infected cells usually occurs over many years, and is associated with random integration of viral DNA into the host genome and specific gene mutations (Zapatka et al., 2020).

## The Microanatomy of the Cervix Determines Prevalence of Premalignant Transformation

**Figure 1:**
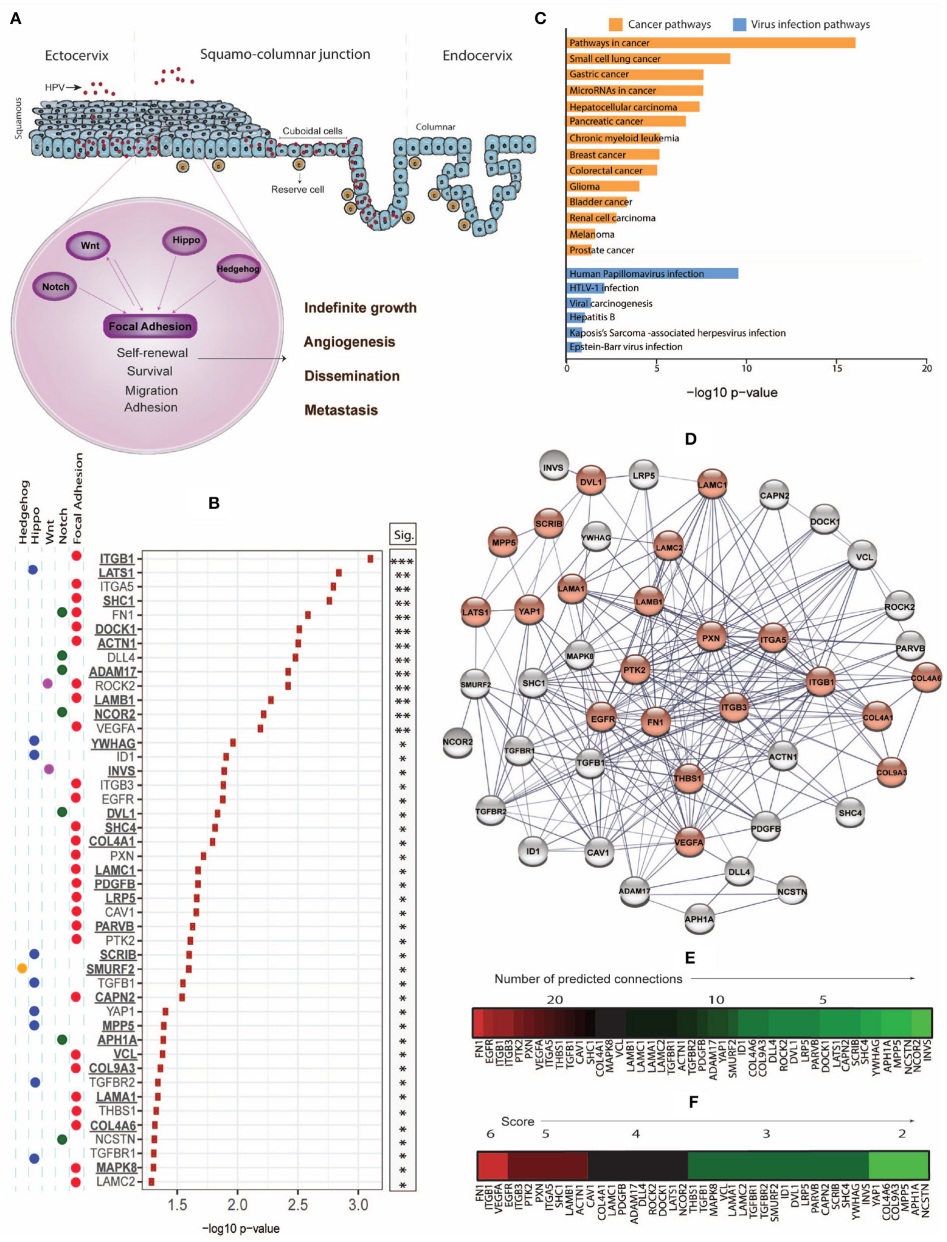
**(A)** While the productive life cycle of HPV occurs in the squamous ectocervix, cervical intraepithelial neoplasia (CIN) and invasive cervical cancer dominantly arise from the squamo-columnar junction (SJL) of the cervix, which contains reserve and cuboidal cells with stem-like properties. Stemness-related pathways such as Focal Adhesion, Notch, Wnt, Hippo, and Hedgehog with cross-talk mechanisms facilitate cell survival, proliferation, epithelial mesenchymal transition, dissemination, and metastasis. **(B)** Using the Xenabrowser online portal (https://xenabrowser.net/) (Goldman et al., 2019), The Cancer Genome Atlas Cervical Squamous Cell Carcinoma and Endocervical Adenocarcinoma (TCGA-CESC) database was filtered on primary site samples (*n* = 304) and screened for significant association of high expression of stemness-related genes with poor 5-year (1,825 days) survival using Kaplan-Meier Plots, comparing subjects of the top and bottom 25% of gene expression. The web-based interactive Xenabrowser analysis integrates data of the following datasets: https://tcga.xenahubs.net/download/TCGA.CESC.sampleMap/HiSeqV2.gz (gene expression data), https://tcga.xenahubs.net/download/survival/CESC_survival.txt.gz (survival data) and https://tcga.xenahubs.net/download/TCGA.CESC.sampleMap/CESC_clinicalMatrix (clinical data). Input genes (*n* = 526) were selected from KEGG pathways (hsa04150, hsa04330, hsa04310, hsa04390, hsa04340). *P*-values of significantly (*p* < 0.05) associated genes where overexpression associated with poorer survival were –log10 transformed and plotted. Underlined genes are those not previously associated with cervical cancer prognosis. **(C)** Significant genes from **(B)** were analyzed for enriched cancer and viral infection pathways using Enrichr and the KEGG 2019 human database. *P*-values were –log10 transformed and plotted. **(D)** STRING-db analysis of genes from **(B)** was used to estimate protein-protein interaction networks and levels of connectivity. Genes enriched in the KEGG HPV infection pathway (hsa05165) are displayed in red. **(E)** Heat map of genes ordered based on highest to lowest connectivity estimated by STRING-db. **(F)** A combined score was established based on the sum of a connectivity score (1: 0–10 connections; 2: 11–20 connections; 3: 21–30 connections) and a *p*-value score (1: –log10 < 1.5; 2: –log10 = 1.5–2.0; 3: –log10 > 2.0), and displayed as heat map.

The cervix can be anatomically divided into the columnar endocervix and the squamous ectocervix, which are connected by the squamo-columnar junction (SCJ) within the transformation zone (Herfs et al., 2017; Huang and Rofstad, 2017). The SCJ is a monolayer consisting of two types of specialized epithelial progenitor cells, reserve cells, and cuboidal cells, with stem-like properties, which as undifferentiated basal cells regenerate the endo- and ectocervix (Figure 1A) (Schiffman et al., 2016). Productive α-HPV infection occurs across the genital mucosa, but malignant transformation and cancer is more common in the cervix, and specifically in the SCJ (Herfs et al., 2011, 2012; Doorbar and Griffin, 2019).

In contrast, the SCJ/transformation zones of vulva, vagina, and anus are differentiated and multi-layered, and the cancer ratio of cervical compared to vulvar/vaginal/anal is ~20:1 while the frequency of HPV infection in cervix and anus is similar (Yang et al., 2015), indicating that HPV infection to the cervical SCJ is more likely to result in malignant transformation compared to other anogenital sites. In the oropharynx, the majority of HPV16 associated cancers arise in the tonsillar crypts and base of tongue (Westra, 2012; Morbini et al., 2015; Doorbar and Griffin, 2019). The specialized porous reticulated epithelium of the tonsillar crypts appears to be more susceptible to carcinogenesis due to the presence of a basal cell type with reduced polarization which results in an increased likelihood for persistent infection, a main risk factor for malignancy (Tomaic, 2016).

## Cervical SCJ Cells Represent a Privileged Site for HPV Infection

Increased vulnerability of SCJ cells to HPV infection and persistence is likely caused by multiple factors. As the cervical SCJ consists of a monolayer of cells, no physical or chemical trauma is required to acquire access to basal cells for infection. Additionally, due to monthly pH fluctuations associated with menstrual hormonal changes, the cervical mucosa undergoes regular changes in the extracellular environment. Also, the cervical SCJ contains an altered composition of host-defense peptides. Alpha defensins have been shown to inhibit HPV infection (Buck et al., 2006). However, SCJ cells and neoplastic lesions lack expression of the human defensin 5 (HD5) (Hubert et al., 2014), and while HD5 doesn't block HPV entry into cells, it redirects HPV viral particles to the lysosome for degradation, therewith counteracting successful infection (Wiens and Smith, 2017). Tetraspanin has been implicated in post-endocytic trafficking of HPVs by interacting with HPV L1, enabling capsid disassembly (Grassel et al., 2016). Interestingly, tetraspanin is overexpressed in SCJ cells (Herfs et al., 2012), possibly enhancing HPV reproduction. Two cytokeratins, Krt7 and Krt9, are also overexpressed in cervical and anal SCJs, and have been shown to enhance translation of HPV transcripts (Kanduc, 2002; Favia et al., 2004). These mechanisms might collectively help establish infection of HPV within SCJ cells more effectively.

In addition to the above, the SCJ favors HPV persistence as it is an immune-privileged site. The density of professional antigen-presenting cells (APCs) in the cervical SCJ and in HPV associated neoplastic lesions is decreased, and this might be related to the lack of HD5, which acts as a chemoattractant. Furthermore, elevated TGF-β levels inhibit E-cadherin expression, which disrupts APC adhesion and subsequently alters APC maturation and regulator T cell polarization (Jiang et al., 2007; Herfs et al., 2008). Prostaglandin E2 is overexpressed in the cervical SCJ and in neoplastic lesions, and inhibits APC migration. Prostaglandin E2 also promotes immune tolerance by stimulating IL-10 and inhibiting IL-12 production (Herfs et al., 2009). RANKL is also overexpressed in SCJ and promotes development of tolerogenic APCs (Demoulin et al., 2015). Also the density of plasmacytoid DCs (pDCs) and regulatory T cells increases from the ectocervix to metaplasia and (pre)neoplasia, and pDCs exposed to HPV-transformed keratinocyte cell lines were inhibited in maturation and IFN-α secretion, and induced regulatory T cells (Demoulin S. et al., 2015). In a transgenic mouse model expressing HPV16 E7 under the control of the keratinocyte-specific K14 promotor and which displays epithelial hyperplasia and shares a gene signature with human cervical intraepithelial neoplasia (CIN) (Tuong et al., 2018), we observed multiple immune-suppressive mechanisms including alteration of antigen-presenting cells and induction of regulatory T cells (Mittal et al., 2013; Chandra et al., 2016; Bashaw et al., 2017, 2019). Together, these observations might explain why the SCJ epithelium expressing viral oncoproteins is more likely than elsewhere in the genital tract to establish persistent HPV infection and malignant transformation.

## Evidence for a Cervical Cancer Stem Cell Niche

The morphology of cervical neoplastic lesions is heterogenous. Well-differentiated squamous lesions tend to arise towards the ectocervix, while lesions with an immature phenotype locate towards the endocervix. However, the unique morphology and genetic signature of cervical SCJ cells is evident in cervical squamous, columnar, and adenosquamous neoplastic lesions, suggesting that most cervical malignancies arise from multipotent cervical SCJ cells (Herfs et al., 2012, 2017; Sato et al., 2017). Further, Krt7+ CIN stage 1 located in the SCJ has a higher risk to progress to cancer than CIN stage 1 located in the ectocervix (Paquette et al., 2016; Mills et al., 2017). SCJ cells have, therefore, been proposed as cervical cancer stem cells (CCSCs). CCSCs can be isolated based on the expression of cytokeratins and CDs and are resistant to cisplatin-chemotherapy and radiotherapy (Mendoza-Almanza et al., 2019). As discussed earlier, cytokeratin expression is increased in CIN and cervical cancer and is associated with metastatic processes (Wang et al., 2006; Ikeda et al., 2008). Biomarkers currently accepted for classification of CCSCs include CD44, CD133, CD49F, ABCG2, ALDH1, OCT4, OPN, and SOX2 (Huang and Rofstad, 2017). Additionally, high level expression of stem cell markers MSI1, ALDH1, and SOX2 in cervical cancer lesions correlates with poor survival (Hou et al., 2015).

Cancer cells inappropriately maintain self-renewal pathways similar to those found in stem cells (SCs), allowing indefinite proliferation, and understanding how these self-renewal pathways are maintained in cancer has therapeutic implications. Cancer stem cells (CSCs) are found in many malignant diseases and are associated with poor survival (Zhu et al., 2020). CSCs tend to remain quiescent, thereby conferring protection from therapies which target mitotic cells, as well as escaping anti-cancer immune responses. Epithelial-mesenchymal transition (EMT) of CSCs is strongly linked with cancer cell metastasis, in which the proportion of CSCs is greatly elevated compared to primary tumors (Aktas et al., 2009).

To enhance our understanding of stemness as a contributor to prognosis of cervical cancer, here we systematically sought association of the expression of genes from key stemness pathways with cervical cancer survival and found that expression of multiple genes contributing to the central Focal Adhesion pathway correlated with poor survival. In the remainder of this perspective, we will discuss these stemness-associated genes and their potential role in HPV infection and cervical cancer progression.

## Dysregulated Stemness-Associated Gene Expression in Primary Cervical Tumors

CCSCs share signaling pathways with SCs, including the Hedgehog, Notch, Wnt, and Hippo pathways that together control organ size, cell proliferation, adhesion, and apoptosis. Many of these pathways interact with the Focal Adhesion pathway that regulates tissue homeostasis and is involved in malignant transformation and metastasis through disrupted control of proliferation, differentiation, apoptosis, and migration (Figure 1A). These interactions place this pathway as a significant target for cancer therapy (Zhou J. et al., 2019). We examined the correlation between stemness-associated genes of key stemness KEGG pathways and the 5-year survival of cervical cancer patients using the Xenabrowser online data analysis tool (Goldman et al., 2019) filtered on The Cancer Genome Atlas Cervical Squamous Cell Carcinoma and Endocervical Adenocarcinoma (TCGA-CESC) dataset of primary tumors (*n* = 304), of which 93% have previously been determined to be HPV positive (Lu et al., 2019). Of 526 analyzed genes, we identified 45 genes where the top quartile of expression level, when compared with the bottom quartile of expression, was associated with poorer 5-year survival (Figure 1B). We also identified 31 genes where the bottom quartile of expression, when compared with the top quartile of expression, correlated with poor survival (data not shown).

To explore potential candidates for targeted therapies, we focused on and henceforth discuss the genes where overexpression correlated with poor survival. Overall, the majority of these genes belonged to the Focal Adhesion pathway, but we also identified genes of other stemness-related pathways including Notch, Hippo, Wnt, and Hedgehog, and many of these gene products signal across pathways (Figure 1B). While overexpression of 18 of these genes has been associated with cervical cancer previously (referenced in Table 1), we also identified 27 new prognosis-associated genes (underscored in Figure 1B). We applied a KEGG pathway enrichment analysis using Enrichr, and observed that the 45 genes were significantly overexpressed in multiple cancers including cancers of lung, stomach, liver, pancreas, breast, colorectum, and bladder (Figure 1C). Enrichr pathway analysis further revealed significant gene expression enrichment in the HPV infection pathway (Figures 1C,D) and other viral infection pathways including HTLV-1, Hepatitis B, and EBV (Figure 1C). We assessed protein-protein interactions for the 45 genes using STRING-db which estimated the number of connections of each gene (Figure 1E). We scored genes according to the number of predicted connections and *p*-value, where genes with a high level of connectivity and *p*-value received an overall high score (Figure 1F), as this can potentially guide identification of biomarker or therapeutic targets.

**Table 1:** Known and novel stemness-associated genes which associate with poor 5-year survival in cervical cancer.

**Novel**	**Known**
ITGB1	ITGA5 (Hazelbag et al., 2007)
LATS1	ITGB3 (Gruber et al., 2005)
SHC1	FN1 (Zhou Y. et al., 2019)
DOCK1	DLL4 (Yang S. et al., 2016)
ACTN1	ROCK2 (He et al., 2010)
ADAM17	VEGFA (Yuan et al., 2018)
LAMB1	ID1 (Peng et al., 2016)
NCOR2	EGFR (He et al., 2015)
YWHAG	PXN (Liu et al., 2018)
INVS	CAV1 (Cheng et al., 2015)
DVL1	PTK2 (Oktay et al., 2003)
SHC4	TGFB1 (Trugilo et al., 2019)
COL4A1	YAP1 (He et al., 2015)
LAMC1	TGFBR2 (Cancer Genome Atlas Research Network, 2017)
PDGFB	THBS1 (Kodama et al., 2001; Wu et al., 2004)
LRP5	NCSTN (Sato et al., 2012; Campos-Parra et al., 2016)
PARVB	TGFBR1 (Fang et al., 2018)
SCRIB	LAMC2 (Yang C. et al., 2016)
SMURF2	
CAPN2	
MPP5	
APH1A	
VCL	
COL9A3	
LAMA1	
COL4A6	
MAPK8	

The focal adhesion kinase PTK2, a gene in the core of the Focal Adhesion pathway, and its central activator fibronectin 1 (FN1), as well as upstream integrin subunits ITGB1, ITGB3, and ITGA5, paxillin (PXN), and thrombospondin 1 (THBS1) represented hub nodes next to the common cancer associates VEGFA and EGFR, and all these genes are part of the Focal Adhesion pathway. A range of PTK2 inhibitors are being tested in clinical cancer trials, with 40 studies currently registered (clinicaltrials.gov, 2020), including one study in patients with head and neck cancer. Integrins have been classified as metastasis-promoting genes due to their function in regulating adhesion, proliferation, and migration through cell-cell and cell-matrix interactions (Cooper and Giancotti, 2019). Paxillin contributes to dissemination and metastasis by accumulating in nascent focal complexes in migrating cells (Maziveyi and Alahari, 2017), and thrombospondin 1 regulates cell-matrix interactions by binding to integrins, fibronectins, and laminins, of which we identified LAMA1, LAMB1, LAMC1, and LAMC2 as associated with poor survival. We also identified 3 collagens (COL4A1, COL4A6, and COL9A3) associated with poor survival, and collagens are known to drive tumor budding (Miyake et al., 2017). Because integrins, paxillin, thrombospondin, laminins, and collagens are positioned upstream of multiple signaling cascades which contribute to cancer invasion and metastasis, these represent desired targets to inhibit these pathways. Other genes associated with poor survival and a high connectivity/*p*-value score included SHC1, ACTN1, PDGFB, ADAM17, DOCK1, LATS1, and NCOR1, and overexpression of many of these genes has been described in association with other cancers.

## Interactions Between HPV Viral Proteins and Stemness Associated Proteins

Expression of some of the genes identified above has impact on the HPV viral life cycle. According to our current understanding of α-HPVs, viral gene expression can modulate cellular pathways to either impart basal stem-cell like characteristics to infected cells in the epithelium or infect specialized epithelial stem-cell populations. In any event, the virus interacts with and affects genes and proteins involved in stemness regulation and maintenance. HPV viral gene expression is highly regulated with limited expression in the infected basal epithelial layer where early protein E2 facilitates viral episomal genome partitioning during the division of infected cells. E2 also regulates cell differentiation and has been shown to interact with focal adhesion kinase PTK2B and FN1 (Muller and Demeret, 2012), two of the top scoring stemness pathway genes we identified to be associated with cervical cancer prognosis. Both of these proteins are involved in cellular adhesion, which suggests an early impact of the viral life cycle not only on stemness but also on cohesion of the epithelium perhaps contributing to invasiveness.

During the phase of low HPV viral gene expression, E6 also plays an important role by helping delay the migration of infected epithelial basal cells to the suprabasal layers. Viral genome amplification occurs in the middle epithelial layers, where deregulated expression of E6 and E7 is seen in association with the manifestation of high-grade neoplasia. In addition to suppressing host immune responses, and associating with key cell cycle proteins p53 and Rb, the early HPV viral proteins also bind to other cellular proteins, enabling suprabasal cell cycle entry. As observed in the epithelial basal layers, levels of FN1 and other adhesion proteins are also increased by E6 and E7 expression in suprabasal layers (Hellner et al., 2009). Additionally, E6 expression can interfere with paxillin, affecting the integrity of the epithelium and leading to invasiveness (Estevao et al., 2019). Expression of integrins such as ITGB3 is also upregulated by HPV E6 (Yim et al., 2004), however, the mechanism is yet to be explored. Angiogenic protein VEGF, another top-scoring gene in our analysis, is involved in increasing vascular permeability and is also upregulated by E6 and E7 (Lopez-Ocejo et al., 2000; Li and Cui, 2015). Interestingly, E6 has been further shown to cause degradation of the polarity protein SCRIB (Gheit, 2019), and overexpression of SCRIB is associated with poor cervical cancer prognosis. This observation is not completely surprising as SCRIB overexpression is seen in a number of cancers, with mislocalisation usually favoring a mesenchymal phenotype (Vaira et al., 2011), and perhaps SCRIB overexpression begins in later stages of HPV associated malignant disease.

HPV early proteins also influence cell proliferation. Among the candidate genes identified in Figure 1, YAP1 enhances proliferation and E6 prevents its proteasome-mediated degradation. YAP1 expression in turn promotes EGFR expression and has recently been shown to be essential for HPV-mediated carcinogenesis (He et al., 2015). In addition to indirect regulation of EGFR expression by E6, EGFR expression is also directly controlled by the early viral protein E5 and promotes genome amplification in mid-epithelial layers (Ilahi and Bhatti, 2020).

E5, E6, and E7 have previously been shown to co-operate to drive tumorigenesis. Co-expression of E5 with either E6 or E7 promotes transformation to a greater extent than with either oncoprotein alone (Ilahi and Bhatti, 2020). The association of these viral proteins with stemness-associated genes suggests a potential for a combined targeted therapy approach against cervical cancers.

## Conclusion

The biggest challenges in the treatment of HPV-mediated cervical malignancy are associated with acquired local immune-suppression, and disruption of cellular homeostatic processes leading to impaired focal adhesion and break-down of extracellular matrix, promoting cancer cell invasion, dissemination, and metastasis. The cancer stem cell theory suggests that these processes are more likely to occur when pluripotent cells with active stemness signaling pathways undergo transformation. HPV infects mucosal epithelial cells at many different anatomical sites, including distinct sub-anatomic sections of the cervix, but malignant transformation occurs predominantly at the SCJ, which contains unique basal cells with stem-like properties, whereas the productive HPV life cycle occurs mainly in the squamous section of the cervix where no undifferentiated cells are seen. The cervical SCJ/transformation zone microanatomy is unique compared to those of other HPV-infected anogenital regions including vagina, vulva, and anus, and this specialized undifferentiated cervical SCJ is associated with a higher risk for malignant transformation. In our analysis of stemness-pathway genes in association with cervical cancer prognosis, we confirmed 18 genes known to be dysregulated in cervical cancer cells. We also identified 27 novel genes whose overexpression significantly associated with poor survival and a majority of these play a role in the central Focal Adhesion pathway. Furthermore, some of these genes were also associated with the HPV infection pathway, and viral protein interactions with stemness proteins have been demonstrated previously.

Importantly, the data presented here indicates but does not prove a direct contribution of the cervical stem cell niche to cervical cancer survival outcomes, as it was collected from bulk RNA sequencing data of primary tumor biopsies. An alternative interpretation would be that cervical cancers which overexpress genes associated with the Focal Adhesion pathway have further progressed on a trajectory towards invasion and dissemination, leading to poorer survival outcomes. Nevertheless, the pathway overlap with HPV infection confirms importance of stemness associated genes in HPV driven cervical cancer progression and might prove important for cancer therapy target selection. While all drivers of HPV infection and malignant transformation are still to be determined, treatment of cervical, and other HPV-associated cancers may be improved by a holistic approach targeting multiple cancerogenic pathways, as well as by initiating a strong HPV-specific immune response through immunization-based immunotherapy that enhances virus-specific immunity (Frazer and Chandra, 2019).

## Data Availability Statement

Publicly available TCGA-CESC datasets were analyzed in this study. This data can be found here: https://xenabrowser.net/.

## Ethics Statement

Ethical review and approval was not required for the study on human participants in accordance with the local legislation and institutional requirements. Written informed consent for participation was not required for this study in accordance with the national legislation and the institutional requirements.

## Author Contributions

MB and JC conceived the work, analyzed data, and wrote the first draft of the manuscript. SL supervised data analysis and revised the manuscript. SP and IF wrote and revised the manuscript. All authors contributed to the article and approved the submitted version.

## Conflict of Interest

The authors declare that the research was conducted in the absence of any commercial or financial relationships that could be construed as a potential conflict of interest.
